# Neutrophil-to-Lymphocyte and Platelet-to-Lymphocyte Ratios as Predictors of Survival and Metastasis for Recurrent Hepatocellular Carcinoma after Transarterial Chemoembolization

**DOI:** 10.1371/journal.pone.0119312

**Published:** 2015-03-05

**Authors:** Wenzhe Fan, Yingqiang Zhang, Yu Wang, Xuehua Yao, Jianyong Yang, Jiaping Li

**Affiliations:** 1 Department of Interventional Oncology, the First Affiliated Hospital of Sun Yat-Sen University, Guangzhou, People’s Republic of China; 2 Department of Medical Imaging, the First Affiliated Hospital of Sun Yat-Sen University, Guangzhou, People’s Republic of China; Yonsei University College of Medicine, KOREA, REPUBLIC OF

## Abstract

**Purpose:**

To evaluate whether neutrophil-to-lymphocyte ratio (NLR) and platelet-to-lymphocyte ratio (PLR) predict survival and metastasis in patients after transarterial chemoembolization (TACE) for recurrent hepatocellular carcinoma (RHCC).

**Materials and Methods:**

Clinical and laboratory data from 132 RHCC patients treated with TACE from January 2003 to December 2012 were retrospectively reviewed. Prognostic factors were assessed by multivariate analysis, and the predictive values of NLR and PLR for overall survival (OS) and extrahepatic metastases were compared.

**Results:**

Pretreatment mean NLR and PLR were 3.1 and 137, respectively. The 0.5-, 1-, and 2-year OS rates were 93.7%, 67.1%, and 10.1% in the low NLR group and 81.1%, 18.9%, and 3.8% in the high NLR group, respectively (*P* = 0.017). The corresponding OS rates in the low and high PLR groups were 92.5%, 58.1%, and 9.7% and 84.6%, 23.1%, and 2.6%, respectively (*P* = 0.030). The discriminatory performance predicting 1-year survival probability was significantly poorer for NLR (area under the curve [AUC] = 0.685, 95% confidence interval [CI] 0.598–0.763) than for PLR (AUC = 0.792, 95% CI 0.712–0.857; *P* = 0.0295), but was good for both ratios for predicting post-TACE extrahepatic metastasis. Multivariate analysis indicated that high PLR (hazard ratio [HR] = 0.373, 95% CI = 0.216-0.644, *P* < 0.001, vascular invasion (HR = 0.507, 95% CI = 0.310–0.832, *P* = 0.007), and multiple tumors (HR= 0.553, 95% CI = 0.333–0.919, *P* = 0.022) were independent prognostic factors for OS.

**Conclusions:**

High NLR and PLR were both associated with poor prognosis and metastasis in RHCC patients treated with TACE, but high PLR was a better predictor of 1-year OS. High PLR, vascular invasion, and multiple tumors were independent, unfavorable prognostic factors.

## INTRODUCTION

Hepatocellular carcinoma (HCC) is the fifth most common cancer and the third leading cause of cancer-related death in the world [1, 2]. Surgery is the treatment of choice for early-stage HCC. However, the intrahepatic recurrence rate is as high at about 70% within 5 years of surgery [3, 4]. The past decade has witnessed the development of various minimally invasive therapies for recurrent hepatocellular carcinoma (RHCC). Among them, transarterial chemoembolization (TACE), a widely used treatment for patients with unresectable RHCC, has shown a demonstrated survival benefit [5, 6]. Although several factors have been identified as prognostic indicators for patients with HCC, including tumor size, vascular invasion, extrahepatic spread, and serum α-fetoprotein (AFP) level [7, 8], these factors have not been validated in unresectable RHCC. Thus, the survival of patients with unresectable RHCC treated with TACE cannot be predicted effectively.

Recent attention has focused on the systemic inflammatory state as a surrogate marker of tumor biology in patients with multiple solid tumors. The neutrophil-to-lymphocyte ratio (NLR) and the platelet-to-lymphocyte ratio (PLR) have been shown to predict overall and postoperative survival in both gastric cancer and non-small-cell lung cancer [9–11]. Recently, clinical studies in advanced HCC have demonstrated that an elevated NLR or PLR, possibly reflecting an inflammatory process elicited by cancer cells, is associated with unfavorable clinicopathologic features [12–14]. However, the prognostic value of these two factors after resection for RHCC has been unclear. The purpose of this study was to evaluate the prognostic performance of NLR and PLR in patients with RHCC treated with TACE.

## MATERIALS AND METHODS

### Ethics Statement

The protocol of this retrospective study was approved by our Institutional Review Board of the First Affiliated Hospital of Sun Yat-Sen University. Written informed consent was obtained from patients prior to treatment.

### Patients

Inclusion criteria for this study were age 18–75 years, cytological/histological diagnosis after hepatectomy of HCC and diagnosis of RHCC by biopsy or two imaging techniques showing typical features of HCC, Eastern Cooperative Oncology Group performance status 0–1, liver function Child-Pugh class A or B cirrhosis, no extrahepatic metastases, and TACE as the sole first-line anticancer treatment for RHCC.

The criteria excluding patients from resection or ablation treatment were multiple tumors, major tumor size greater than 5 cm, presence of tumor thrombi in the first portal branch or main portal vein, and inability of the remaining liver to tolerate surgery or ablation.

Exclusion criteria for the study were loss to follow-up within three months after treatment, portal vein tumor thrombi with complete main portal vein occlusion and without adequate collateral circulation around the occluded portal vein, hepatic decompensation (including ascites), esophageal or gastric variceal bleeding or hepatic encephalopathy, severe underlying cardiac or renal diseases, severe coagulation disorders (prothrombin activity < 40% or a platelet count < 50×10^9^/L), or active infection at the time of blood sampling to establish NLR and PLR.

Between January 2003 and December 2012, 437 patients with RHCC underwent TACE after hepatectomy at our institution. However, 225 patients were excluded from the current study because they did not meet the inclusion criteria. Among them, 94 patients received other treatments (percutaneous ablation in 63, resection in 15, chemotherapy in 11, and liver transplantation in 5), 45 patients had an ECOG score ≥ 2, 32 patients had extraheptic metastasis, 28 patients were class C Child-Pugh C, 19 patients had a platelet count < 50×10^9^/L, and 7 patients had complete main portal vein occlusion. An additional 63 patients were excluded due to incomplete laboratory data. Finally, 17 patients were lost to follow-up, resulting in a final enrollment for this study of 132 patients.

### Data Recording

Clinical and laboratory data were collected from all patients prior to TACE. Clinical data included patient age, sex, symptoms, hepatectomy history, and Child-Pugh score. In addition, imaging data were collected based on abdominal computed tomography (CT) or magnetic resonance imaging (MRI), including tumor size, number, and macrovascular invasion. Laboratory data included white blood cell count (WBC), neutrophil count, lymphocyte count, platelet count (PLT), hepatitis B surface antigen (HBsAg), α-fetoprotein (AFP), albumin (ALB), total bilirubin (TBil), prothrombin time (PT), alanine aminotransferase (ALT), and aspartate aminotransferase (AST). The NLR or PLR was defined as the ratio derived from division of the neutrophil or platelet count by the lymphocyte count. Patients were stratified into high or low NLR and PLR groups based on the mean levels of NLR and PLR within seven days prior to the first TACE [13].

### TACE Procedures

TACE was performed using techniques previously described [15, 16]. Visceral angiography was performed to assess the arterial blood supply of the liver after introduction of a selective catheter. The same three chemotherapeutic agents with the same dosage were used consistently in this study, regardless of tumor number and size. Hepatic artery infusion chemotherapy was performed using 300 mg carboplatin (Bristol-Myers Squibb, New York, NY, USA). Subsequently, chemolipiodolization was performed using 50 mg epirubicin (Pharmorubicin; Pfizer Inc., New York, NY, USA) mixed with 5 ml of lipiodol (Lipiodol Ultra-Fluide; André Guerbet Laboratories, Aulnay-sous-Bois, France). If residual flow remained after infusion of these agents, additional lipiodol was injected. In large tumors for which we could not achieve stasis in a tumor-feeding artery with the maximum amount of iodized oil (20 ml), embolization was performed with absorbable gelatin sponge particles (Gelfoam; Hangzhou Bi-Trumed Biotech Co., Ltd., Hangzhou, Zhejiang, China) 350–560 μm in diameter. The injection was slowed or discontinued if reflux occurred. Patients were observed carefully after treatment, and analgesia was administered when necessary.

### Follow-up

At follow-up, AFP levels, liver function, white blood cell counts, and platelet counts, were measured as well as imaging studies, were performed including triphasic liver CT scans and chest radiography during the first month after TACE. The same liver CT scans and chest radiography were performed every three months for the first year following the procedure and every six months thereafter. Liver MRI or biopsy was performed to define suspicious lesions revealed by CT or an elevated AFP. If necessary, chest CT, bone scintigraphy, positron emission tomography (PET), and biopsy were also performed for the diagnosis of metastasis and/or recurrence. When residual tumor or new recurrence was diagnosed, performance of a second TACE was considered using the same criteria as those used at the time of the initial TACE. If TACE was contraindicated, administration of sorafenib or palliative treatment was considered. Overall survival (OS) and occurrence of extrahepatic metastasis were recorded. The last follow-up date for this study was December 2013.

### Statistical Analysis

The analysis software used was SPSS for Windows version 18.0 (SPSS Inc., Chicago, IL, USA) and MedCalc for Windows version 11.0 (MedCalc Software, Ostend, Belgium). Continuous variables were expressed as the mean ± standard deviation (SD) and compared using Student’s *t* test. Categorical data were presented as frequency and were analyzed using the Pearson χ^2^ test or Fisher’s exact test. Survival curves were estimated using Kaplan-Meier analyses, and the differences in survival rates between groups were compared using the log-rank test. Univariate analysis was performed to assess significant differences in clinicopathologic characteristics that influence overall survival after TACE. Multivariate analysis was performed using Cox regression analysis for significant variables identified by univariate analysis. To estimate the predictive value of NLR and PLR for OS and metastasis, receiver operating characteristic (ROC) curves were constructed using MedCalc. Additionally, the area under the receiver operating characteristic curve (AUC) was calculated to compare the discriminatory performance of NLR or PLR for the prediction of OS and metastasis using the z-statistic [17, 18]. All statistical tests were two-sided and differences were considered significant with a *P* < 0.05.

## RESULTS

### Demographic Data

This study included 132 patients with RHCC who had undergone TACE during the study period, for whom complete data were available. The study group was composed of 87 men (65.9%) and 45 women (34.1%). The median age was 49 years (range, 23–75 years). The median values and ranges of white blood cell, neutrophil, lymphocyte, and platelet counts, as well as PLR and NLR, of the patients prior to undergoing TACE are shown in Table 1. The mean pretreatment NLR was 3.1. Accordingly, patients with NLR < 3.1 were assigned to the low NLR group, while patients with NLR ≥ 3.1 were assigned to the high NLR group. The mean pretreatment PLR was 137. Patients with PLR < 137 were assigned to the low PLR group, while patients with PLR ≥ 137 were assigned to the high PLR group. There were no significant differences in demographic and clinicopathologic features between the low and high groups for either NLR or PLR (Table 2).

**Table 1:** Values for total white blood cells, neutrophils, lymphocytes, platelet counts, platelet-to-lymphocyte ratios, and neutrophil-to-lymphocyte ratios in recurrent hepatocellular carcinoma patients (n = 132).

Blood components	Mean	Median	Minimum	Maximum	Normal values
**Total white blood cells** (×10^9^/L)	5.75±2.05	6.89	3.61	14.58	4.00–10.00
**Absolute neutrophil count** (×10^9^/L)	4.07±2.00	3.67	1.42	8.18	1.80–6.40
**Absolute lymphocyte count** (×10^9^/L)	1.68±0.80	1.46	0.30	4.99	1.00–3.30
**Neutrophil-to-lymphocyte ratio**	3.08±2.02	2.02	0.34	8.05	-
**Total platelets** (×10^9^/L)	195.53±58.23	177	78.00	381.00	100–300
**Platelet-to-lymphocyte ratio**	137.34±71.11	107	54.02	438.76	-

**Table 2:** Comparison of clinicopathologic and demographic features between patients with low NLR and high NLR expressed as mean ± SD or n.

Factor	Low NLR (<3.1), n = 79	High NLR (≥3.1), n = 53	*P*-value	Low PLR (<137), n = 93	High PLR (≥137), n = 39	*P*-value
**Sex:** men/women	50/29	37/16	0.279	61/32	26/13	0.536
**Age (y):** ≤50/>50	52/27	34/19	0.494	65/28	21/18	0.109
**WBC** (×10^9^/L)	6.27±1.51	6.46±2.13	0.390	6.27±1.51	6.46±2.13	0.390
**PLT** (×10^9^/L)	165.38±54.21	166.76±55.45	0.601	160.93±50.72	176.48±58.65	0.167
**ALT** (U/L)	43.95±23.24	45.42±27.02	0.740	43.95±23.24	45.42±27.02	0.740
**AST** (U/L)	45.81±23.61	51.54±26.49	0.195	45.81±23.61	51.54±26.49	0.195
**HBsAg:** +/-	61/18	39/14	0.391	72/21	28/11	0.317
**AFP (ng/ml):** ≤400/>400(ng/ml)	21/58	22/31	0.089	25/68	18/21	0.042
**Child-Pugh classification:** A/B	66/13	47/6	0.287	78/15	35/4	0.279
**TBil** (μmol/l)	17.29±8.69	18.48±9.38	0.464	17.29±8.69	18.48±9.38	0.464
**ALB** (g/l)	38.6±6.95	38.2±3.98	0.670	38.6±6.95	38.2±3.98	0.670
**PT** (s)	12.4±1.97	13.1±2.62	0.147	12.4±1.97	13.1±2.62	0.147
**Tumor number:** <3/≥3	22/57	16/37	0.460	28/65	10/29	0.454
**Major lesion size:** <3 cm/≥3 cm	43/36	23/30	0.143	53/40	13/26	0.011
**Vascular invasion:** +/-	26/53	20/33	0.264	29/64	15/24	0.270

SD = standard deviation, NLR = neutrophil-to-lymphocyte ratio, PLR = platelet-to-lymphocyte ratio, WBC = white blood cells, PLT = platelets, ALT = alanine transaminase, AST = aspartate transaminase, HBsAg = hepatitis B surface antigen, AFP = α-fetoprotein, TBil = total bilirubin, ALB = albumin, PT = prothrombin time, ALB = albumin.

### Overall Survival

**Fig 1:**
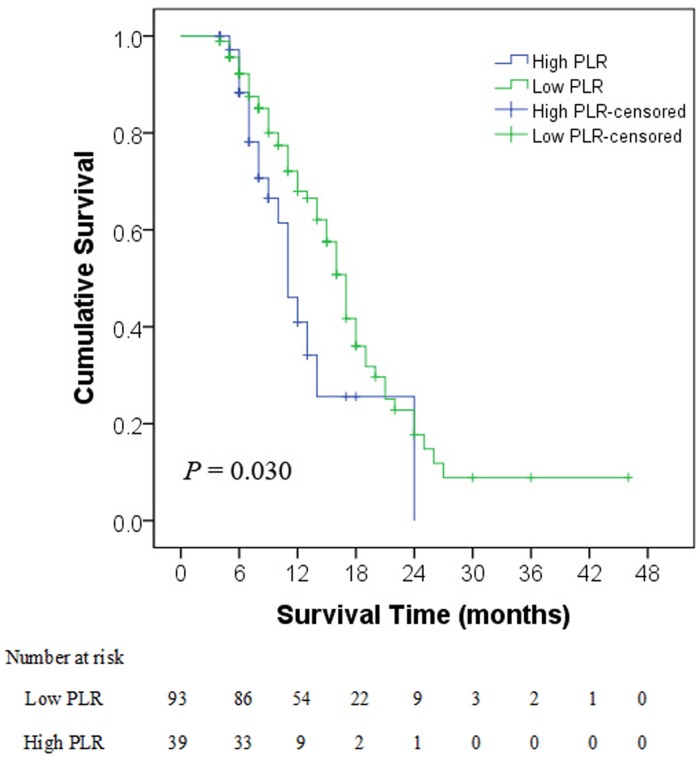
Overall survival curves in patients with low and high NLR. The difference between groups were statistically significant (log-rank test, *P* = 0.017). PLR, platelet-to-lymphocyte ratio; NLR, neutrophil-to-lymphocyte ratio.

**Fig 2:**
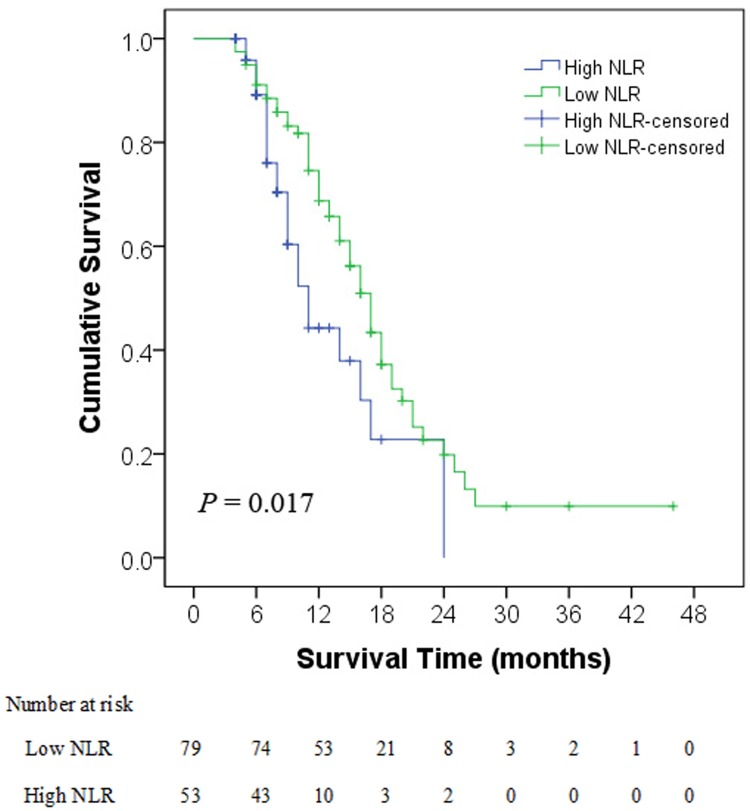
Overall survival curves in low and high platelet-to-lymphocyte ratio patients. The difference between groups were statistically significant (log-rank test, *P* = 0.030). PLR, platelet-to-lymphocyte ratio; NLR, neutrophil-to-lymphocyte ratio.

At the median follow-up of 11 months (range, 4–46 months), 58 patients (43.9%) remained alive. The 0.5-, 1-, and 2-year OS rates for all patients were 88.6%, 46.2%, and 7.5%, respectively. The median survival of patients in the high NLR group was 11 months (range, 4–24 months), compared with 17 months (range, 4–46 months) in the low NLR group. The long-term survival was significantly better for patients with low NLR than for patients with high NLR (log-rank test: *P* = 0.017; Fig. 1). Patients with PLR < 137 also had better long-term survival than did patients with PLR ≥ 137 (log-rank test: *P* = 0.030; Fig. 2). The median survival of patients in the high PLR group was 12 months (range, 4–24 months), compared with 17 months (range, 4–46 months) in the low PLR group. Extrahepatic metastases, including pulmonary (29 patients), osseous (11 patients), retroperitoneal lymphatic (8 patients), and intracranial (4 patients), developed in 33 patients by the end of the follow-up period.

### Correlations

Univariate analysis identified the following factors as affecting OS: elevated AFP level (> 400 ng/mL, *P* = 0.003), multiple tumors (≥ 3, *P* = 0.023), large tumor (≥ 3 cm, *P* < 0.001), vascular invasion (*P* < 0.001), high NLR (≥ 3.1, *P* = 0.001), and high PLR (≥ 137, *P* < 0.001). Multivariate analysis identified high PLR (hazard ratio [HR] = 0.373, 95% confidence interval [CI] 0.216–0.644, *P* < 0.001), vascular invasion (HR = 0.507, 95% CI 0.310–0.832, *P* = 0.007), and multiple tumors (HR = 0.553, 95% CI 0.333–0.919, *P* = 0.022) as independent predictors of poor survival (Table 3).

**Table 3:** Cox proportional hazards model of baseline prognosticators for overall survival in 132 patients with RHCC undergoing TACE.

	Univariate			Multivariate		
Characteristics	HR	95% CI	*P*-value	HR	95% CI	*P*-value
**Sex:** men/women	1.045	0.649–1.681	0.857	-	-	-
**Age (y):** ≤50/>50	0.710	0.447–1.127	0.144	-	-	-
**HBsAg:** +/-	0.771	0.459–1.295	0.325	-	-	-
**AFP (ng/ml):** ≤400/>400(ng/ml)	0.481	0.295–0.785	0.003	-	-	0.458
**Child-Pugh classification:** A/B	0.775	0.472–1.274	0.315	-	-	-
**Tumor number:** <3/≥3	0.572	0.354–0.925	0.023	0.553	0.333–0.919	0.022
**Major lesion size:** <3 cm/≥3 cm	0.415	0.256–0.673	<0.001	-	-	0.744
**Vascular invasion:** +/-	0.389	0.240–0.630	<0.001	0.507	0.310–0.832	0.007
**NLR:** <3.1/≥3.1	0.443	0.276–0.711	0.001	-	-	0.130
**PLR:** <137/≥137	0.373	0.216–0.644	<0.001	0.373	0.216–0.644	<0.001

HR = hazard ratio, CI = confidence interval, RHCC = recurrent hepatocellular carcinoma, TACE = transarterial chemoembolization, HBsAg = hepatitis B surface antigen, AFP = α-fetoprotein, NLR = neutrophil-to-lymphocyte ratio, PLR = platelet-to-lymphocyte ratio.

**Fig 3:**
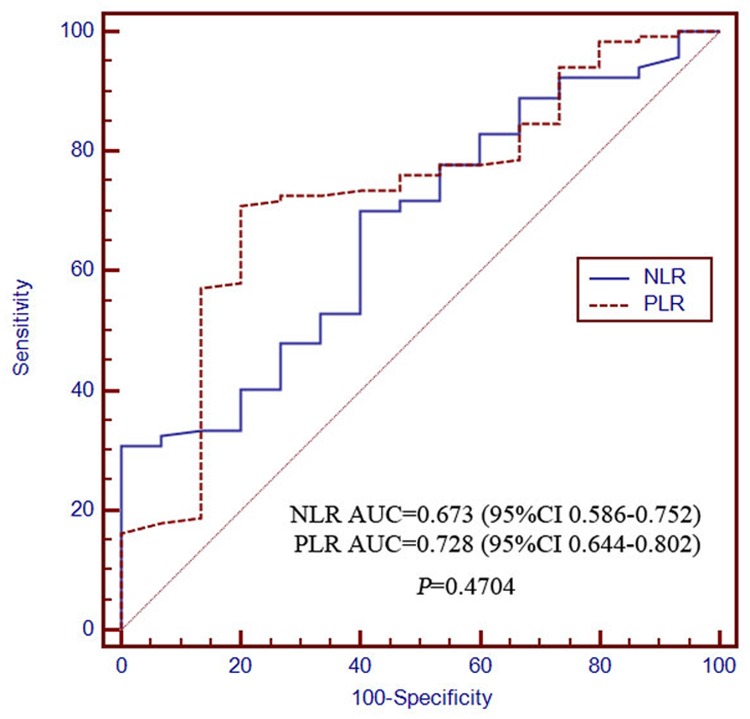
The receiver operating characteristics curves for 0.5-year overall survival in NLR and PLR patients. The difference between groups were not statistically significant (*P* = 0.4704). PLR, platelet-to-lymphocyte ratio; NLR, neutrophil-to-lymphocyte ratio.

**Fig 4:**
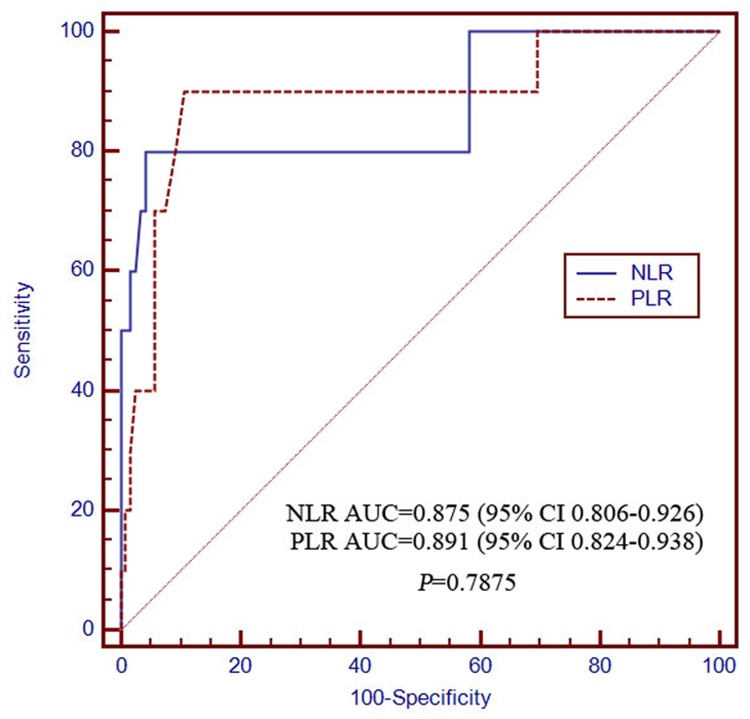
The receiver operating characteristics curves for 2-year overall survival in NLR and PLR. The difference between groups were not statistically significant (*P* = 0.7875). PLR, platelet-to-lymphocyte ratio; NLR, neutrophil-to-lymphocyte ratio.

**Fig 5:**
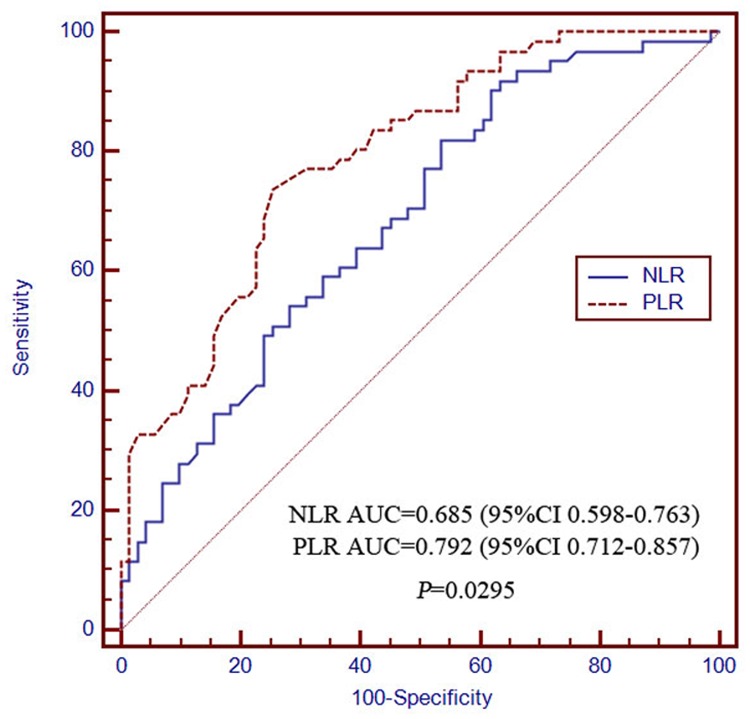
The receiver operating characteristics curves for 1-year overall survival in NLR and PLR patients. The difference between groups were statistically significant (*P* = 0.0295). PLR, platelet-to-lymphocyte ratio; NLR, neutrophil-to-lymphocyte ratio.

**Fig 6:**
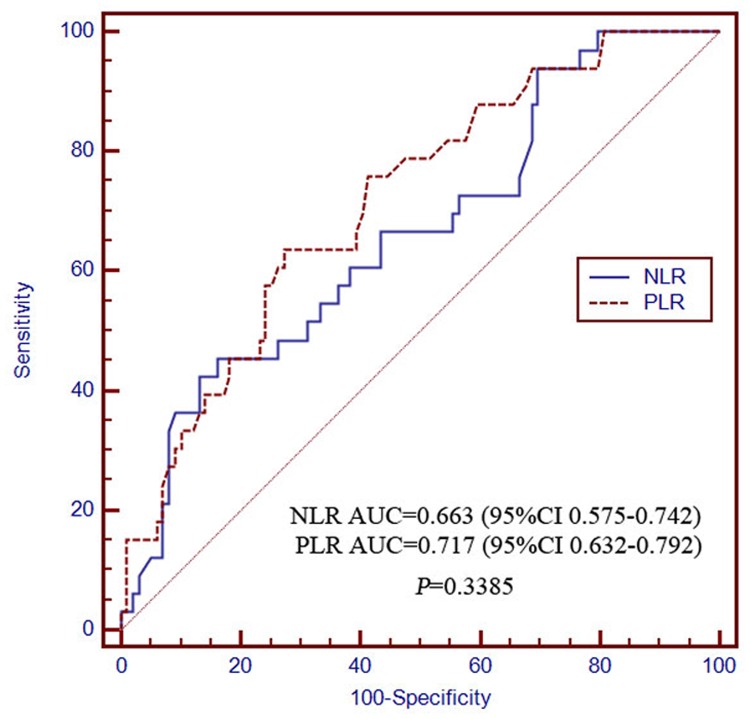
The receiver operating characteristics curves for extrahepatic metastasis NLR and PLR. The difference between groups were not statistically significant (*P* = 0.3385). PLR, platelet-to-lymphocyte ratio; NLR, neutrophil-to-lymphocyte ratio.

The AUCs of the NLR for determining 0.5- and 2-year survival were 0.673 (95% CI 0.586–0.752) and 0.875 (95% CI 0.806–0.926), respectively, which were not significantly different from those of the PLR at 0.728 (95% CI 0.644–0.802) and 0.891 (95% CI 0.824–0.938), respectively (Figs. 3 and 4). The discriminatory performance in predicting 1-year survival probability was significantly poorer for NLR (0.685, 95% CI 0.598–0.763) than for PLR (0.792, 95% CI 0.712–0.857; *P* = 0.0295; Fig. 5). The discriminatory performance for predicting post-TACE extrahepatic metastases was good for both NLR (AUC = 0.663, 95% CI 0.575–0.742, *P* = 0.0031) and PLR (AUC = 0.717, 95% CI 0.632–0.792, *P* < 0.0001; Fig. 6).

### Complications

There were no treatment-related deaths. The major complication (upper gastrointestinal bleeding) rate was 0.01% (1 of 132 patients). Abdominal pain, fever, and vomiting were observed in 93.9%, 83.3%, and 51.5% of all patients, respectively. All patients recovered from complications following treatment of symptoms in one to seven days.

## DISCUSSION

Inflammation has been found to play an important role in the pathogenesis and progression of malignant tumors, including HCC [9–13, 19–21]. A recent study described a lymphocyte-dependent host tumor-immune response [22]. Inflammation promotes tumor angiogenesis, invasion, and metastasis through a subset of regulatory T lymphocytes and chemokines. Therefore, patients with subset-specific lymphocytopenia may have a higher risk of tumor recurrence and a worse prognosis [23, 24]. In HCC patients, increased infiltration of CD4^+^ T lymphocytes at the tumor margins has been reported to be associated with a lower recurrence rate and better prognosis [25].

Neutrophils can be stimulated to express various cytokines, such as interleukin 8 (IL-8), which is the core of the inflammatory and immune responses and results in modifications to the microenvironment supportive of tumor progression and development [26]. Neutrophils have also been shown to promote tumor growth and metastasis by secreting vascular endothelial growth factor, angiopoietin-1, and matrix metalloproteinase-9 [27]. The angiogenic activity resulting from neutrophilia could provide tumors with a survival advantage, leading to increased recurrence rates and decreased survival in HCC patients.

In general, a higher platelet count indicates less less fibrotic burden, that is, a less chance of de-novo HCC development and hepatic decompensation. However, a previous study focusing on platelet count demonstrated that thrombocytosis was associated with more advanced disease and inoperable cancer, as well as being an independent prognostic factor for epithelial ovarian cancer [28]. Furthermore, a large glycoprotein secreted by platelets in the blood, thrombospondin-1 (TPS-1), promotes tumor cell adhesion, migration, invasion, and angiogenesis, thereby potentiating tumor progression [29]. As reported, PLR was associated with some unfavorable clinico-pathologic features in malignant tumors [19–21, 28, 29], which may have greater influences on the survival in HCC patients.

Many clinical studies have demonstrated that elevation of systemic inflammatory markers, such as neutrophils, platelets, lymphocytes, NLR or PLR, is associated with unfavorable clinicopathologic features in HCC [12, 13, 30]. However, while these studies focused on the prognostic capability of markers of inflammation in HCC treated by surgical resection [12], transplantation [31], ablation [32], or TACE [13], none evaluated these markers in resected RHCC post-TACE or explored the relationship between NLR and PLR. Gomez et al. [12] determined the NLR in 96 patients scheduled for HCC resection and found that the median disease-free survival was eight months in cases in which NLR was ≥ 5, compared with 18 months when NLR was < 5. After analyzing the outcomes of 101 patients with hepatitis B virus-associated HCC who received liver transplants, Wang et al. [31] concluded that elevated preoperative NLR significantly increased the risk of recurrence and that patients with both NLR ≥ 3 and more than three tumors were not good candidates for liver transplantation. Another study found that NLR > 3.2 was associated with worse overall survival in early-stage HCC patients treated with radiofrequency ablation [32]. Huang et al. [13] demonstrated that a high NLR (≥ 3.3) independently predicted poor survival in patients with unresectable HCC undergoing TACE, while an increased postoperative NLR indicated a better outcome for patients following TACE. In addition, a recent study calculated PLR in 150 HCC patients without treatment limitations and reported that an elevated PLR was associated with worse OS [30]. The results of the current study are consistent with previous studies demonstrating that high pretreatment NLR and PLR are predictors of poor survival and, further, that PLR is an independent prognostic factor for patients with RHCC after resection treated with TACE.

A study comparing the predictive value of NLR and PLR in HCC patients undergoing liver transplantation found that the NLR was the best predictor of dropout and the last PLR had an intermediate statistical ability to predict tumor recurrence after liver transplantation [33]. Patients with an NLR value > 5.4 had poor 5-year intention-to-treat (ITT) survival rates, while PLR was found to better stratify patients in relation to tumor-free survival. The current study evaluated the ability of NLR and PLR to predict survival of RHCC patients treated with TACE. We found that both PLR and NLR had predictive value for 0.5-, 1-, and 2-year survival rates in patients with RHCC. When AUC results were compared in a subgroup of patients, there was no significant difference in the prediction of 0.5-year survival rates between NLR and PLR, although PLR was a better predictor of 1-year survival than NLR. In addition, while the predictive value of PLR was similar to that of NLR for 2-year survival in the current study, the number of patients who survived ≥ 2 years was too small (n = 10) to achieve robust statistical power. Therefore, additional studies are needed to determine whether PLR provides extra predictive value, with good performance and agreement, for the long-term survival of patients with RHCC. In addition, given the complex mechanisms underlying the host immune response to a malignant tumor, it is likely that the relationship between NLR, PLR, and development of RHCC will be the subject of further research.

To our knowledge, this is the first study to evaluate the ability of NLR and PLR to predict the risk of extrahepatic metastases in RHCC. Our results demonstrate that NLR and PLR were strongly linked to the risk of distant metastases. High NLR and PLR may reflect inflammation, which can promote tumor cell adhesion, migration, and invasion, as well as angiogenesis. These are well-known risk factors for HCC metastasis [26–29].

In addition, we demonstrated that AFP level, tumor number, tumor size, and vascular invasion affected OS and that multiple tumors, vascular invasion, and a high PLR were independent unfavorable prognostic factors. These results are consistent with those of previous reports, indicating that these tumor characteristics are associated with a more aggressive phenotype [2, 13, 30, 32]. This reinforces the importance of meticulous surveillance protocols for high-risk patients.

The limitations of the current study arise from factors that could affect the NLR and PLR of patients with RHCC before the performance of TACE. First, the host’s immune status may be different in patients with HCC who are infected with hepatitis B compared to those with HCC who are not infected with hepatitis B. Although no previous studies have reported hepatitis B-associated changes in NLR or PLR, this factor might have introduced heterogeneity into our study. Second, levels of blood neutrophils and platelets could be reduced by cirrhosis-associated hypersplenia and TACE-associated myelosuppression. Because the chemotherapeutics were injected into the artery supplying the tumor, TACE has very small effect on the bone marrow. A more rigorous research design could eliminate these limitations. Another limitation is that 305 patients were excluded, which may influence the generalizability of the study results. Finally, although previous studies have validated the prognostic capability of NLR or PLR in HCC treated by surgical resection [12], transplantation [31], ablation [32], and TACE [13], the utility of these markers should be further validated and compared to treatment-naive patients. Further prospective studies are needed to confirm and expand our preoperative prognostic score model for patients with RHCC.

In conclusion, the current clinical study demonstrated that high NLR and PLR are associated with poor prognosis and extrahepatic metastases in patients with RHCC treated with TACE. Multiple tumors, vascular invasion, and a high PLR were identified as independent unfavorable prognostic factors. High PLR had a better discriminatory performance than NLR for predicting 1-year OS of patients with RHCC. Further studies in larger and more homogeneous populations are needed to validate the prognostic capability of NLR and PLR in RHCC patients undergoing TACE.
